# Molecular Research and Treatment of Skin Diseases

**DOI:** 10.3390/ijms23105435

**Published:** 2022-05-13

**Authors:** Ulrich Koller, Johann W. Bauer

**Affiliations:** EB House Austria, Research Program for Molecular Therapy of Genodermatoses, Department of Dermatology and Allergology, University Hospital of the Paracelsus Medical University Salzburg, 5020 Salzburg, Austria; joh.bauer@salk.at

The intention of this Special Issue is to highlight current treatment options to target the cause, as well as disease-associated complications, of skin diseases, including a group of monogenetic skin disorders referred to as genodermatoses. The clinical heterogeneity of genodermatoses is reflected by primary disease manifestations including skin blistering and scaring, mucosal involvement, tumorigenesis, increased photosensitivity and dermal vulnerability. The high diversity of associated genes and disease-causing mutations results in a variety of phenotypic severities and clinical outcomes. Therefore, our special issue has the focus on causal to symptom-relieving approaches, which include gene, RNA, and cell therapies, as well as drug developments based on small molecules. The monogenetic disease epidermolysis bullosa (EB) represents a group of genodermatoses, in which various pathogenic gene mutations are causal for the disease phenotype [1]. The main primary manifestations in EB include blister formation on the skin and mucous membranes upon minimal mechanical stress induction. The severity of the disease and the corresponding subtype depend on the affected gene, the mutation type, and the mode of inheritance. Current treatment strategies are limited to symptomatic relief, such as wound care and blister prevention, as causal treatment options are still at the preclinical stage.

Therefore, in our Special Issue, one focus lays on the targeting of the severe recessive dystrophic variant of EB (RDEB), in which mutations within *COL7A1* are causal for the disease. In RDEB, painful erosions, debilitating scarring, and the development of aggressive squamous cell carcinoma early in life are serious disease manifestations. Gene replacement therapies comprise the most clinically advanced approaches, showing potential in the junctional form of EB in particular [2,3,4]. Here, upon viral transduction of the “wild-type” transgene into patient keratinocytes, genetically corrected epidermal sheets are grafted onto patients. The resultant long-lasting regenerated stable skin is associated with an increased quality of life for the treated patient [3]. Gene replacement strategies based on viral transgene delivery always bear the risk of genomic toxicity and initial applications of a retroviral-based cDNA replacement therapy for dystrophic EB had limited success [5]. Thus, the main challenge with ex vivo, as well as in vivo gene therapies for dystrophic EB is to improve delivery of transgenes into skin cells. One way to address this issue is the combined usage of polymeric-based non-viral delivery systems and minicircle DNA as developed by the group of Wenxin Wang in Dublin [6]. Aiming at the replacement of the full-length *COL7A1* cDNA, human epidermal keratinocytes were treated with various minicircle vector systems expressing type VII collagen (C7). The comparison of different promoters revealed a high C7 expression especially with the eukaryotic translation elongation factor 1 α (EF1α) promoter and a *COL7A1*-tissue-specific promoter, achieving comparable levels to normal human wild-type keratinocytes. In summary, the authors describe a promising non-viral topical treatment option for C7 restoration in RDEB patients, with a high safety profile and that is adaptable to other genetic conditions in the future [6].

As an alternative to gene targeting, RNA-based therapies are emerging as powerful alternatives to treat genodermatoses. One RNA therapy option is based on antisense-oligonucleotide (ASO)-mediated exon skipping, as reviewed by Vermeer and colleagues in the course of this special issue [7]. Here, short ASOs are used to remove the mutant exon from the pre-messenger RNA, leading to the production of a slightly truncated version of the absent protein with restored function within the affected skin. The authors summarized the preclinical advances made in exon skipping for EB over the last 15 years and include a discussion on the prospects and current challenges of this RNA-based therapy approach. Alternatively, Mayr et al. used the RNA *trans*-splicing technology, also known as Spliceosome-Mediated RNA *trans*-splicing or SMaRT, to repair mutations within the gene *COL7A1* at the pre-mRNA level [8]. Therefore, an RNA *trans*-splicing molecule, termed RTM, was designed capable of accurately replacing *COL7A1* exons 1–64 in an endogenous setting. An increase in wild-type transcript and protein levels was the consequence of the retroviral RTM delivery into immortalized RDEB keratinocytes. Notably, immunofluorescence staining of skin equivalents, derived from RTM-treated keratinocytes, revealed the accurate deposition of restored C7 within the basement membrane zone between epidermis and dermis. In summary, the authors present for the first time a 5′ *trans*-splicing approach for *COL7A1* reprogramming at pre-RNA level, showing promise in reverting the RDEB-associated phenotype towards wild-type and thus addressing an urgent need of this patient population [8]. Besides RNA repair, the RNA *trans*-splicing technology can be further used for cancer cell targeting and killing, as recently described by Woess et al. [9]. Although chemo- and radiotherapies represent a highly effective way to kill cancer cells, these therapeutic options are associated with serious side effects. Thus, Woess et al. describe in their study a promising approach based on RNA *trans*-splicing to target and kill tumor cells while leaving normal cells unharmed. An RNA *trans*-splicing molecule (RTM) specific for the cancer target gene *Ct*-*SLCO1B3*, carrying the suicide gene *HSV1*-thymidine kinase, was introduced into SCC cells isolated from patients with RDEB. Accurate *trans*-splicing between the RTM and endogenous *Ct-SLCO1B3* transcripts resulted in the functional activation of *HSV1-tk* upon ganciclovir (GCV) treatment. Notably, systemic treatment of mice bearing RTM-expressing cancer cells led to a significant reduction of tumor weight and volume in comparison to respective controls, highlighting the potential to use RNA *trans*-splicing for skin cancer therapy in EB [9].

SCCs from patients with recessive dystrophic EB are highly aggressive and are the leading cause of death among RDEB patients. However, in general, SCCs with different etiologies display molecular similarities. In order to select drugs for repurposing to treat life-threatening SCCs, Zauner et al. investigated the similarities in transcriptomes between more frequent head and neck SCCs and SCCs from organ transplant recipients and RDEB patients [10]. Here, the aim of this in silico approach was the assumption that SCC-derived transcriptome profiles mirror critical tumor pathways. Differentially expressed genes in SCCs were then used to mine drug-perturbation data, allowing the authors to identify drugs showing potential in the treatment of SCCs in RDEB.

Besides the therapeutic treatment of genodermatoses, other skin-related topics were addressed in our special issue, for instance the potential antiaging effect of topical collagen tripeptides in the skin [11]. It is known that glycation of the skin is associated with skin aging, especially in skin exposed to environmental factors. Previous studies revealed that hydrolyzed collagen tripeptides (CTP) have anti-inflammatory and antiaging effects although the exact mechanism is unclear. Thus, Lee et al. studied the effect of CTP on facial skin via a single-arm study including 22 Asian women. At week four, skin wrinkles, elasticity, and density were improved, and a reduction in skin accumulation of advanced glycated end products was observed. Therefore, the authors assume that a topical CTP application might prevent clinical aging phenotypes via the inhibition of glycation and oxidative stress resulting in a delay of cellular aging [11].

Montero et al. analysed the influence of the chemotherapeutic agent paclitaxel on the skin especially in primary keratinocytes [12]. As a result, they showed that paclitaxel impairs different cellular processes in primary keratinocytes and in a 3D epidermal model. Observed alterations include an increased release of the inflammatory cytokines IL-1 α, IL-6 and IL-8, the production of reactive oxygen species (ROS) and apoptosis, and a reduced endothelial tube formation in dermal microvascular endothelial cells (HDMEC). The presented study helps in understanding the impact of the drug paclitaxel on the skin. One other study discusses the epigenetic characteristics of cutaneous T-cell lymphomas and current status of research on epigenetic-targeted therapies against these malignant diseases [13]. Nakanishi et al. analysed in their study the connection of malnutrition and inflammatory skin diseases [14]. Therefore, they used a mouse model of dermatitis to analyse the pathophysiology of malnutrition in inflammatory skin conditions, as well as the efficiency of possible treatment options. They conclude that active control of skin inflammation is necessary to prevent gastrointestinal manifestations.

In summary, our special issue comprises a broad spectrum of topics with a main focus on therapeutic options for the blistering skin disease epidermolysis bullosa. Here, targeting of the primary cause of the disease as well as the severe secondary manifestation in form of SCC development stand in the foreground. Furthermore However, other themes deal with skin aging, malnutrition in inflammatory skin diseases, epigenetics of cutaneous T-Cell lymphomas and the impact of the therapeutic agent paclitaxel on the skin, thus expanding the readership to various disciplines in dermatology.
